# The Acute Effects of Milk Consumption on Systemic Inflammation after Combined Resistance and Plyometric Exercise in Young Adult Females

**DOI:** 10.3390/nu14214532

**Published:** 2022-10-28

**Authors:** Emily C. Fraschetti, Lauren E. Skelly, Joel L. Prowting, Ali A. Abdul-Sater, Andrea R. Josse

**Affiliations:** 1 School of Kinesiology and Health Science, Faculty of Health, York University, Toronto, ON M3J 1P3, Canada; 2 Department of Kinesiology, Faculty of Applied Health Sciences, Brock University, St. Catharines, ON L2S 3A1, Canada

**Keywords:** dairy products, inflammation, acute exercise, exercise-induced muscle damage

## Abstract

High-intensity/impact exercise elicits a transient increase in inflammatory biomarkers. Consuming nutrient-dense wholefoods, like milk, following exercise may modulate post-exercise inflammation and aid recovery. We examined the effect of post-exercise skim milk consumption (versus an isoenergetic, isovolumetric carbohydrate [CHO] drink) on acute exercise-induced inflammation in untrained females. Using a randomized crossover design, 13 healthy females (age = 20 ± 2.3 y; BMI = 21.0 ± 1.1 kg/m^2^) completed two bouts of combined resistance/plyometric exercise followed by either skim milk (MILK) or CHO at 5-min and 1 h post-exercise. Serum interleukin [IL]-1β, IL-6, IL-10, and tumor necrosis factor-alpha (TNF-α) concentrations were measured at pre-exercise, 15-min, 75-min, 24 h, and 48 h post-exercise. IL-6 increased 15-min post-exercise vs. all other timepoints (time effect, *p* = 0.017). Between 24 and 48 h, IL-10 decreased and increased in the MILK and CHO conditions, respectively (interaction, *p* = 0.018). There were no significant effects for IL-1β or TNF-α. Relative concentrations of IL-1β (*p* = 0.049) and IL-10 (*p* = 0.028) at 48 h post-exercise were lower in MILK vs. CHO. Milk post-exercise did not influence the absolute concentration of pro-inflammatory cytokines; however, there were divergent responses for the anti-inflammatory cytokine, IL-10, and milk reduced the relative inflammatory response at 48 h (vs. CHO) for IL-1β and IL-10. This demonstrates the potential for milk to modulate inflammation post-exercise in this sample.

## 1. Introduction

Following acute high-intensity exercise, exercise-induced muscle damage (EIMD) can occur [1] and is typically followed by a transient inflammatory response. This is characterized by increases in both pro- and anti-inflammatory cytokines which act as messenger molecules that attract and activate leukocytes (white blood cells) to the damaged muscle/tissue to facilitate repair and regeneration [2]. However, this inflammatory response may result in additional untargeted (“secondary”) damage to the surrounding tissue (i.e., muscle), primarily caused by infiltration of neutrophils to the site of damage which subsequently produce/release reactive oxygen species (ROS) and proteases [2] that may delay complete recovery. It has been posited that attenuating the post-exercise inflammatory response may reduce secondary damage thus alleviating the severity of EIMD and improving recovery time [3]. Many strategies have been employed for reducing EIMD, including stretching, pharmacology (i.e., anti-inflammatory drugs), massage, cryotherapy, and nutrition (i.e., vitamin/mineral supplementation, antioxidants, protein) [4].

Consuming milk following exercise is an established nutritional strategy for enhancing rehydration and glycogen resynthesis and for stimulating muscle protein synthesis during acute recovery periods, likely related to the immediate provision of key nutrients (protein and carbohydrates) involved with these processes [5,6,7]. Milk could also be beneficial for reducing EIMD and attenuating post-exercise inflammation as various components within milk have been identified as having anti-inflammatory or antioxidant potential [8,9,10]. Specifically, key amino acids such as cysteine, histidine, and glycine, found in milk protein can downregulate tumor necrosis factor-alpha (TNF-α)-induced activation of nuclear factor-kappa B (NF-κB) in human monocytes [11]. Calcium supplementation has also been shown to reduce systemic cytokine concentrations in mice [12]. Although some human studies report no benefits, other studies have found that milk consumption improves creatine kinase (CK) response, muscle soreness, muscle function, and performance during recovery [13]. Given the anti-inflammatory properties of milk, modulation of the acute inflammatory response may be one mechanism for the reported improvements in these outcomes.

Two human studies have assessed flavoured milk intake and inflammation following acute exercise. Both found no effect of flavoured milk (strawberry or chocolate; vs. carbohydrate and water controls) on multiple systemic cytokines following high-intensity exercise in trained [14] and untrained [15] populations; however, the use of flavoured milk (which contains added sugars), may have masked a potential anti-inflammatory effect of milk, as foods with higher glycemic indices may increase postprandial inflammation and oxidative stress [16]. Few studies have directly examined white milk (herein referred to as milk) intake (vs. an isoenergetic carbohydrate drink) and inflammation following acute exercise, but these studies were done in trained individuals and only examined one inflammatory biomarker, C-reactive protein (CRP) noting neutral [17,18] or negative effects [19] of milk despite improvements in performance and strength. Whether post-exercise milk consumption influences pro- and anti-inflammatory cytokines following high-impact exercise in untrained females remains unknown. Therefore, this study aimed to characterize and compare the acute (up to 48 h) systemic response of a range of pro- and anti-inflammatory cytokines (tumor necrosis factor-alpha (TNF-α), interleukin-1 beta (IL-1β), interleukin-6 (IL-6), and interleukin-10 (IL-10)) following a single session of intense, muscle-damaging, high-impact/load (resistance and plyometric) exercise in combination with the post-exercise consumption of either skim milk or an isoenergetic, isovolumetric, carbohydrate control drink in young, normal weight, adult females. Specifically, we examined absolute concentrations of circulating cytokines over 48 h, and the relative change at the completion of the trials (i.e., at 48 h). We hypothesized that relative to carbohydrate, milk would attenuate the pro-inflammatory response while enhancing the anti-inflammatory response to exercise, and attenuate markers of inflammation by the end of the acute trial (i.e., at the 48 h timepoint).

## 2. Materials and Methods

This study is a secondary analysis of a previously published project [20] that examined bone biomarker responses following post-exercise milk and carbohydrate consumption. This research study was approved by both Brock (#17-402) and York University’s (#2019-405) Human Research Ethics Boards and was registered at www.clinicaltrials.gov (NCT03615989).

### 2.1. Participants

Thirteen females (age: 20.3 ± 2.3 y; body mass index [BMI]: 21.0 ± 1.1 kg/m^2^) were recruited from two universities (York and Brock; Toronto and St. Catharines, ON, Canada) and provided written informed consent prior to participating in the study. Participants were healthy, untrained/recreationally active, and not currently engaged in a resistance training program, and had no known dairy protein allergy or lactose intolerance [20].

### 2.2. Study Design

Using a crossover design, participants completed two trials in a randomized order: (1) exercise + carbohydrate (CHO), and (2) exercise + milk (MILK). Prior to the trial, participants underwent a familiarization exercise session to determine their estimated 1 repetition maximum on a series of exercise machines, as previously described [20]. Following randomization, participants reported to the lab after an overnight fast for a rested blood sample, followed by participation in the acute, high-intensity exercise protocol, consisting of plyometric and resistance exercise (which included several sets of repeated eccentric contractions likely inducing muscle damage [21]), under the supervision of a certified trainer. Following exercise, participants consumed their first trial drink within 5–10 min, which was then immediately followed by a post-exercise blood sample (15-min post-exercise). Drinks consisted of either 550 mL of unflavoured skim milk (MILK; 200 kcal, 29 g carbohydrates, 20 g protein, 0 g fat) or an isoenergetic, isovolumetric, carbohydrate drink (CHO; 52 g maltodextrin and 550 mL water; 208 kcal, 52 g carbohydrates, 0 g protein, 0 g fat). Calorie-free sweetener was added to the CHO drink to improve palatability. A second, identical, trial drink was consumed 1 h after consumption of the first trial drink, again followed by a blood sample (75-min post-exercise). Participants returned to the laboratory 24 h and 48 h post-exercise for the final two blood samples. Additional details about the exercise and supplement protocol have been published elsewhere [20]. In terms of menstrual cycle consistency, for those who were naturally cycling (n = 8), trials were separated by a minimum 4-week washout and occurred during the early follicular phase of the menstrual cycle [20]. For those on monophasic hormonal birth control (n = 5), trials were separated by a minimum 2-week washout and occurred during hormone delivery [20].

Participants were asked to keep their diet as consistent as possible across both trials and completed a two-day food diary starting the day of the exercise trial. Dietary intakes were analyzed using the ESHA Food Processor Program (Food Processor SQL, ESHA Research, Salem, OR, USA).

### 2.3. Blood Sample Collection and Biochemical Analysis

Venous blood samples were collected from a vein in the antecubital fossa of each participant by trained study personnel using a standardized venipuncture technique at pre-exercise, 15-min, 75-min, 24 h, and 48 h post-exercise. Following each draw, blood samples rested at room temperature for 25 min before being centrifuged (1300× *g* for 15 min at 4 °C). Aliquots of serum were stored at −80 °C until analysis.

Serum concentrations of IL-6, TNF-α, IL-1β, and IL-10 were analyzed by *Eve Technologies* (Calgary, AB, Canada) in duplicate using microbead multiplex assay kits (Human high sensitivity T cell panel HSTCMAG-28SK, Millipore Corp, Burlington, MA, USA). Average coefficients of variation for each cytokine were 8.7% (IL-6), 5.6% (TNF-α), 7.5% (IL-1β), and 8.0% (IL-10).

### 2.4. Statistical Analysis

Data were assessed for normality by examining skewness and kurtosis z-score and non-normally distributed data were log transformed (IL-6, TNF-α, and IL-10). One participant was unable to complete a 48 h blood draw during their CHO trial, so the last obtained measure (24 h post-exercise) was carried forward. One participant’s serum IL-6 values were >3 standard deviations above the mean and were removed from the analysis. Two-way repeated-measures ANOVA (RM-ANOVA; 2 within factors: time and trial) were conducted on the absolute concentrations of each cytokine to assess main effects and interactions. The Greenhouse Geisser correction factor was used when sphericity was violated. Following a significant main effect for time or interaction, post hoc analyses (paired t-tests) were conducted to detect differences over time and between groups. One-tailed paired t-tests were used to compare relative percent change (to pre-exercise) values at 48 h between CHO and MILK. Significance for all tests was set at *p* < 0.05. Statistical analyses were completed using SPSS version 27.0 (SPSS, Chicago, IL, USA).

## 3. Results

### 3.1. Dietary Intake

There were no differences in dietary intakes between conditions when nutrient analyses were performed without the trial drinks added (to reflect background/habitual dietary intakes; Table 1).

### 3.2. Absolute Concentrations of Cytokines

Absolute cytokine concentrations at each timepoint are shown in Table 2. The concentration of IL-6 increased at 15-min post-exercise (+0.21 pg/mL; post hoc *p* = 0.001) and returned to pre-exercise at 75-min with no differences between conditions. For IL-10, there was a significant time x trial interaction. In MILK, IL-10 was elevated at 15-min compared to 75-min (+0.40 pg/mL; post hoc, *p* = 0.04), and IL-10 decreased from 24 h to 48 h (−0.41 pg/mL; post hoc, *p* = 0.01). In CHO, IL-10 increased from 24 h to 48 h (+0.61 pg/mL; post hoc, *p* = 0.04), and 24 h was lower than 15-min (−0.95 pg/mL; post hoc, *p* = 0.04). There were no main effects or interactions for the absolute concentrations of TNF-α and IL-1β.

### 3.3. Relative Change in Cytokine Concentrations at 48 h Post-Exercise

**Figure 1:**
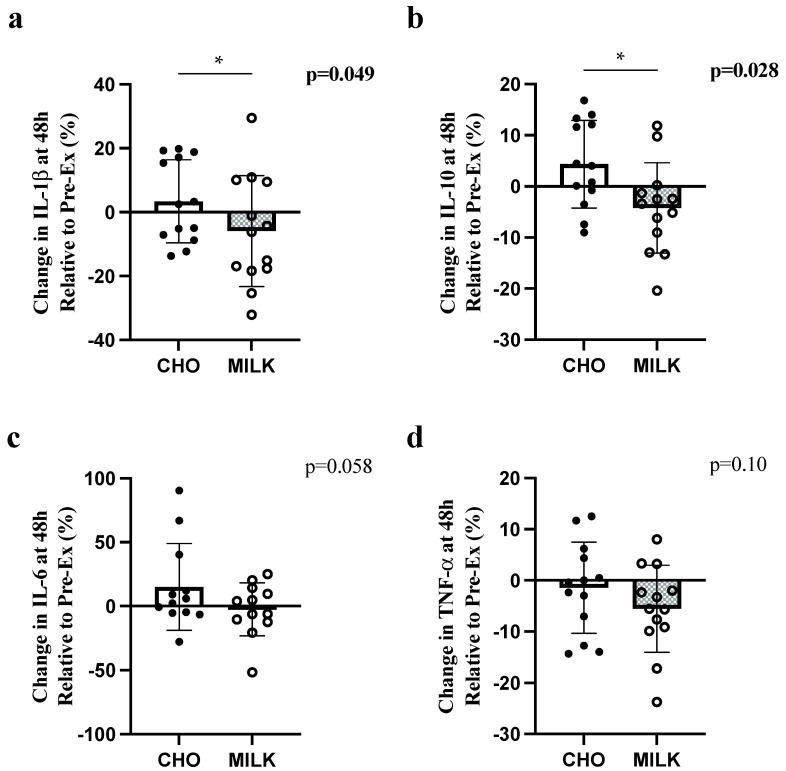
Comparison of the percent change (to pre-exercise) in IL-6 (**a**), TNF-α (**b**), IL-1β (**c**) and IL-10 (**d**) at 48 h post-exercise in the CHO and MILK trials. Symbol (*) denotes a significant difference between trials for one-tailed *t*-tests (*p* < 0.05). Data are presented as mean ± standard deviation. Circles represent individual participant changes. IL-1β: interleukin-1 beta; IL-10: interleukin-10; IL-6: interleukin-6; TNF-α: tumor necrosis factor-alpha.

There were significant differences between conditions in the percent change from pre-exercise to 48 h for IL-1β and IL-10 (Figure 1a,b, respectively), with greater relative decreases following MILK vs. CHO. There were no significant differences in relative concentration for IL-6 or TNF-α (Figure 1c,d, respectively).

## 4. Discussion

This study aimed to investigate the influence of milk vs. an isoenergetic CHO beverage on markers of inflammation following an acute high-intensity/impact muscle-damaging exercise bout. We observed an influence of post-exercise nutrition on the inflammatory response following exercise. Specifically, we observed divergent responses in the absolute concentration of the anti-inflammatory cytokine IL-10 between 24 and 48 h. In MILK, IL-10 decreased at 48 h compared to 24 h, whereas in CHO, IL-10 increased between these timepoints. In contrast, there was no influence of nutrition on the absolute concentrations of any pro-inflammatory cytokine (IL-6, TNF-α, and IL-1β). We also demonstrated a significant difference in the relative change at 48 h between the trials, with a greater decrease in the relative concentration of IL-10 and IL-1β in MILK compared to CHO. These results may indicate a potential benefit of milk consumption on attenuating markers of inflammation post-exercise.

The greater relative decrease in IL-10 in MILK compared to CHO at 48 h may reflect a negative effect of milk, when IL-10 is assessed in isolation, as a lower relative concentration of IL-10 may indicate a more pro-inflammatory state. However, due to the complex interaction between different cytokines, it is prudent to interpret this finding in the context of extended inflammatory networks [9]. Further, IL-10 production is induced by NF-κB activation, which also regulates many pro-inflammatory cytokines [22], thus a reduction in NF-κB activation would result in both lower pro-inflammatory cytokine and IL-10 production. Indeed, the relative concentration of other pro-inflammatory cytokines were also lower in the present study at 48 h (significantly lower in IL-1β and trending lower in IL-6) during MILK (vs. CHO). This may, in part, be related to the provision of amino acids within milk, specifically, cysteine, histidine, and glycine, as these amino acids have been shown to reduce TNF-α-induced activation of NF-κB in human monocytes [11]. As anti-inflammatory cytokines often rise to oppose the inflammatory cascade [9], these findings may indicate the earlier completion of the inflammatory response to exercise and a potential benefit of milk at 48 h for reducing inflammatory indices. This may help speed acute recovery compared to CHO, but more research is needed to confirm this finding.

The inflammatory response following damaging-exercise can generally be divided into two phases: a pro-inflammatory phase, focused on clearing damaged muscle tissue, followed by an anti-inflammatory phase that is integral for muscle repair and regeneration [23]. IL-10, an anti-inflammatory cytokine, is a key signaling molecule for the onset of this secondary anti-inflammatory phase and it initiates macrophage phenotype transition on the continuum from M1 to M2 [23]. Therefore, in our study we hypothesize that the increase in IL-10 in CHO at 48 h (vs. CHO at 24 h) may indicate the onset of muscle repair and generation (i.e., the anti-inflammatory phase), whereas within MILK the onset of muscle repair occurred earlier (noting the decline in IL-10 between 24 and 48 h). These results, in conjunction with our findings of the reduced relative concentration of IL-10 and IL-1β at 48 h, may illustrate a benefit of post-exercise milk consumption in speeding recovery from muscle damage. Interestingly, two other studies examining whey protein have also observed an influence of nutrition on IL-10 following exercise [24,25]. One study reported significantly greater absolute increases in IL-10 after whey protein consumption vs. a water control at 8 h post-exercise in competitive adolescent swimmers [25], and another investigation observed higher levels of IL-10 (118% difference; however, *p* > 0.05) at 4 h post-exercise following the consumption of a whey protein cake vs. a carbohydrate control in trained adults [24]. Both studies [24,25] used a component of milk (i.e., whey protein) rather than the wholefood. Wholefood dairy products provide additional bioactive nutrients, such as calcium [26], in addition to whey protein, which may also positively affect the inflammatory response [27]. Indeed, a recent crossover study conducted by members of our group demonstrated an increase in resting (i.e., not the post-exercise response) IL-10 following a 5-day high-intensity soccer training camp in female youth athletes consuming 3 servings/day Greek yogurt vs. isoenergetic carbohydrate pudding [28]. Importantly, our study adds to the literature by demonstrating that milk can influence post-exercise IL-10 in the later stages of the post-exercise response (which was not measured in the above studies). Whether milk also influences the earlier (<8 h) response during mid-stages of recovery remains unknown. Given the findings of these studies and the present investigation, future work comparing the effects of wholefood dairy products vs. their individual anti-inflammatory constituents (e.g., calcium, whey protein) on inflammatory network responses over a comprehensive time course is warranted.

It has been well documented that IL-6 increases immediately in response to exercise [29], which is congruent with the results of the present investigation. The initial rise in IL-6 (i.e., during and immediately following exercise, as observed in the present study) has been hypothesized to come from muscle and elicit an anti-inflammatory effect [29]. Secondary rises in IL-6 have also been shown in mid and late stages of exercise recovery, which may be attributed to release from other sources (i.e., immune cells, such as monocytes) and tend to act in a pro-inflammatory nature [30]. The nutrition and exercise protocol did not alter absolute concentrations of IL-1β or TNF-α. Studies examining the response of TNF-α following exercise have also observed no change [31], which may be due to the suppression of TNF-α by muscle-derived, anti-inflammatory IL-6 [29]. While some investigations have reported increases in IL-1β following highly damaging exercise, the findings are inconsistent [32]. Research shows that IL-1β is produced locally (in the muscle) in response to damaging exercise, however, the changes in systemic concentrations do not align with intra-muscular concentrations indicating that IL-1β may be tightly regulated and not readily secreted systemically [33]. This may explain the lack of change within our study. Lastly, while we did not observe an influence of nutrition on any pro-inflammatory marker, we reported divergent responses on the anti-inflammatory marker IL-10. Thus, our study and others [24,25,28] have collectively demonstrated that post-exercise dairy consumption may be more beneficial in altering the anti-inflammatory environment. It therefore may be prudent to investigate additional anti-inflammatory markers (e.g., IL-4) in future research.

Our study is the first to examine post-exercise unflavoured milk consumption on several inflammatory cytokines. The lower relative concentrations at 48 h in MILK vs. CHO suggests a potential beneficial effect of consuming milk following exercise on acute inflammation. Previous similar studies investigating milk (vs. an isoenergetic carbohydrate control) consumed after different high-intensity interval exercise protocols in trained female athletes have reported equivocal results for the acute CRP response [17,18,19]. We sought to improve our understanding of the influence of milk on inflammation, as the evaluation of only one inflammatory marker (in previous studies) severely limits the ability to characterize/understand the intricacies of the inflammatory response. We also chose to examine unflavoured milk as opposed to flavoured milk, as done by others [14,15] because the utilization of ‘flavoured milk’ may mask the anti-inflammatory effects of milk due to the higher sugar content and glycemic index [16]. However, we did not have sampling times between 75-min and 24 h post-exercise which may have aided in understanding the divergent responses observed in IL-10. Given the two-day nature of our study, our sampling protocol was designed to help minimize participant burden (and thus missed intermediate timepoints). Additionally, it may have been beneficial to include a no-energy control (i.e., water consumption post-exercise), to further elucidate the influence of energy alone (i.e., calories) on post-exercise inflammation. Lastly, with the inflammatory response being a secondary analysis, our study was limited in that we did not include direct measures of muscle recovery. Future studies like ours should employ measures of muscle soreness, muscle function and performance to assess the effect of nutrition on EIMD more comprehensively. Despite these limitations, our findings provide crucial initial insight into the characterization of the inflammatory response following the post-exercise consumption of milk.

To better characterize the inflammatory response post-exercise and advance our understanding of how dairy nutrition can alter the inflammatory response, additional studies that investigate more complex inflammatory markers are needed. For example, the examination of peripheral blood leukocytes would allow for greater characterization of the inflammatory response and stronger inference about the local immune response, including insight into the different stages of muscle damage and repair [2]. Examining systemic leukocyte populations using flow cytometry would also provide an intricate measure of cell counts and changes within leukocyte subpopulations (e.g., classical, CD14^hi^CD16^low^, and non-classical, CD14^low^CD16^hi^ monocytes) following exercise. Furthermore, measuring intracellular cytokine production of circulating immune cells may aid in determining the source of cytokine release. The ability to determine the source of systemic cytokines following an exercise stressor is especially important when examining IL-6, as the source of IL-6 determines its action (i.e., pro-inflammatory [primarily from immune cells] or anti-inflammatory [primarily from muscle]). Collecting muscle biopsies would also allow for direct measurement of IL-6 and examination of the local leukocyte response (including macrophage phenotypes) and provide direct measurement of muscle damage and the phases of repair and regeneration. Lastly, it is important to assess the post-exercise inflammatory response following the consumption of fermented dairy products, such as yogurt, as fermented products may provide additional benefit to modulating the inflammatory response [34] and have been shown to influence IL-10 following one week of training [28]. These additional measures and comparisons will greatly improve our understanding of the post-exercise inflammatory response and how it can be influenced through post-exercise nutrition to improve recovery from EIMD.

## 5. Conclusions

The present study reports a novel comparison of high-intensity/impact exercise-induced changes in IL-6, TNF-α, IL-1β and IL-10 following the post-exercise consumption of milk or a carbohydrate control drink in young adult females. While there were no differences in the pro-inflammatory cytokine response (IL-6, TNF-α, IL-1β), we observed differences in the anti-inflammatory cytokine IL-10, which warrants further investigation, particularly in the context of recovery following EIMD. Future research should examine additional aspects of the inflammatory response (i.e., systemic and intracellular cytokines, immune cells, tissue cell signalling) and compare wholefoods to their key anti-inflammatory constituents.

## Figures and Tables

**Table 1:** Average daily dietary intake during the MILK and CHO trials, based on analysis of 2-day food records (day of exercise and day post-exercise) excluding the nutrient information from the provided trial drinks.

Dietary Variable	CHO	MILK	*p* Value
Energy intake (kcal)	1757 ± 627	1673 ± 616	0.46
Protein (g)	67 ± 27	61 ± 28	0.09
Carbohydrate (g)	221 ± 91	212 ± 102	0.55
Fat (g)	68 ± 30	68 ± 24	0.97
Vitamin D (IU)	47 ± 54	48 ± 50	0.96
Calcium (mg)	561 ± 374	473 ± 266	0.18
Iron (mg)	10 ± 4	10 ± 6	1.00
Magnesium (mg)	194 ± 145	202 ± 113	0.76
Potassium (mg)	1768 ± 1379	1914 ± 1316	0.35
Selenium (mcg)	60 ± 36	62 ± 33	0.91

Values are mean ± SD; Nutritional contribution of the two CHO beverages: ~410 kcals, 104 g carbohydrate. Nutritional contribution of the two MILK beverages: ~400 kcals, 40 g protein, 58 g carbohydrate, 720 IU Vitamin D, 1200 mg calcium, 1.44 mg iron, 160 mg magnesium, 1680 mg potassium.

**Table 2:** Absolute serum concentration of cytokines at pre-exercise, 15-min, 75-min, 24 h, and 48 h post-exercise in the carbohydrate (CHO) and milk (MILK) trials.

Cytokine (pg/mL)	CHO					MILK					RM-ANOVA *p*-Values		
	Pre-Ex	15-min	75-min	24 h	48 h	Pre-Ex	15-min	75-min	24 h	48 h	Group	Time	Int
IL-6	1.59 (2.15)	1.74 * (2.25)	1.73 (2.27)	1.79 (2.65)	1.63 (2.65)	1.54 (3.66)	1.58 * (3.81)	1.60 (3.59)	1.52 (3.34)	1.32 (3.14)	0.67	**0.017**	0.37
TNF-α	6.81 (3.21)	6.87 (2.93)	6.53 (2.91)	7.01 (2.47)	6.86 (1.76)	6.32 (5.25)	5.65 (4.29)	6.38 (4.95)	6.68 (5.42)	6.53 (5.97)	0.98	0.27	0.81
IL-10	5.25 (3.85)	7.43 ^†^ (4.92)	5.94 (4.03)	5.24 (4.20)	5.46 ^†^ (4.73)	5.74 (2.92)	6.39 ^#^ (1.43)	5.46 (2.40)	5.63 (3.27)	5.24 ^†^ (2.11)	0.76	0.12	**0.018**
IL-1β	1.96 (0.77)	2.00 (0.82)	2.03 (0.82)	1.98 (0.78)	2.00 (0.75)	2.29 (1.16)	2.31 (1.16)	2.22 (1.03)	2.32 (1.18)	2.11 (0.96)	0.31	0.46	0.12

Values are presented as median ± interquartile range (IQR) for log-transformed variables (IL-6, TNF-α and IL-10) and mean ± standard deviation for IL-1β. n = 13 for IL-1β, TNF-α and IL-10, n = 12 for IL-6. Significant *p*-values from the repeated measures ANOVA are presented in bold. Symbols denote a significant difference vs. all other timepoints (*, *p* < 0.05), vs. 75-min (^#^, *p* < 0.05) and vs. 24 h (^†^, *p* < 0.05). IL-6: interleukin-6; TNF-α: tumor necrosis factor-alpha; IL-10: interleukin-10; IL-1β: interleukin-1 beta; Pre-Ex: pre-exercise; Group: main effect of group; Time: main effect of time; Int: time x trial interaction.

## Data Availability

The data sets presented in this article are not readily available. Requests to access the data sets should be directed to A.R.J., ajosse@yorku.ca.
